# Distance in Motion: Response Trajectories Reveal the Dynamics of Number Comparison

**DOI:** 10.1371/journal.pone.0025429

**Published:** 2011-09-23

**Authors:** Seppe Santens, Sofie Goossens, Tom Verguts

**Affiliations:** Department of Experimental Psychology, Ghent University, Ghent, Belgium; Kyushu University, Japan

## Abstract

Cognitive and neuroscientific evidence has challenged the widespread view that perception, cognition and action constitute independent, discrete stages. For example, in continuous response trajectories toward a target response location, evidence suggests that a decision on which target to reach for (i.e., the cognition stage) is not reached before the movement starts (i.e., the action stage). As a result, instead of a straight trajectory to the correct target response, movement trajectories may curve toward competing responses or away from inhibited responses. In the present study, we examined response trajectories during a number comparison task. Participants had to decide whether a target number was smaller or larger than 5. They had to respond by moving to a left or a right response location. Replicating previous results, response trajectories were more curved toward the incorrect response location when distance to 5 was small (e.g., target number 4) than when distance to 5 was large (e.g., target number 1). Importantly, we manipulated the response mapping, which allowed us to demonstrate that this response trajectory effect results from the relative amount of evidence for the available responses across time. In this way, the present study stresses the tight coupling of number representations (i.e., cognition) and response related processes (i.e., action) and shows that these stages are not separable in time.

## Introduction

Imagine you participate in a cognitive experiment. On each trial, you are shown two Arabic digits in the range 1 to 9. Your task is to respond with a left key press if the left stimulus is numerically larger and with a right key press if the right stimulus is larger. How do you perform this number comparison task? Perhaps Arabic digits are first processed visually (i.e., perception) and are then represented as numerical magnitudes on which a decision is made (i.e., cognition). Once this decision is made, a response is executed (i.e., action). Although this division into perception, cognition, and action seems obvious and often goes unquestioned, it is an assumption that may or may not be valid. Cisek and Kalaska [1] recently argued against this assumption by reviewing a large number of neuroscientific findings. They propose instead that any organism is continuously interacting with the environment and that it is not always clear what is to be labeled as perception, cognition, or action in this seamless interaction. One consequence of this so-called “ecological perspective” is that actions are not necessarily the result of a finished cognitive process, but can be influenced by ongoing cognition.

**Figure 1:**
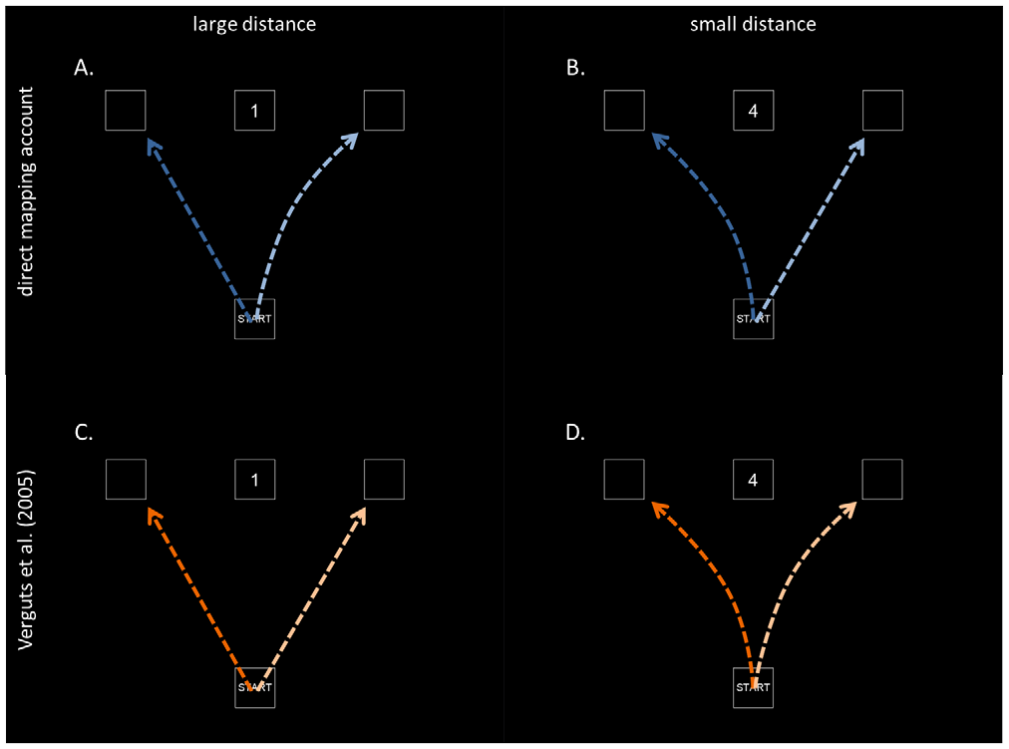
Schematic overview of the predictions. Predictions derived from the direct mapping account (blue arrows) and the Verguts et al. (2005) model (orange arrows). The subject's task is to compare the centrally presented number to 5. Dark (blue or orange) arrows represent the number line congruent mapping “if smaller press left, if larger, press right”; light (blue or orange) arrows represent the number line incongruent mapping “if smaller press right, if larger press left”.

If only the endpoint of an action is registered (i.e. response time of an actual button press), we cannot test whether the action stage is influenced by ongoing cognitive processing. However, different methodologies have been proposed to investigate this. Song and Nakayama [2] reviewed several studies registering continuous response trajectories towards a target response location. The evidence using this technique suggests that the decision on which target to reach for (i.e. the cognition stage) is not reached before the movement starts (i.e. the action stage). As a result, instead of a straight trajectory to the correct target response, movement trajectories can curve towards competing responses (or away from inhibited responses). In one of these studies, Song and Nakayama [3] used a special case of the number comparison task, in which a number has to be compared with the standard 5. Participants had to reach for a left target response if a number smaller than 5 was presented, to a central target response if the number was equal to 5 and to a right target response if the number was larger than 5. For targets other than 5, response trajectories curved more inwards from the straight trajectory for numbers close to 5 compared to numbers far from 5 (see Figure 1, panels A and B, dark blue arrows). In terms of the dependent variable used by Song and Nakayama [3], deviation from the midline increased with increasing numerical distance. Two alternative interpretations for this observation are possible.

With the response mapping used by Song and Nakayama [3] (move to the left if number is smaller than 5, to the right if number is larger than 5), relatively small target numbers resulted in a more leftward response trajectory and relatively large numbers in a more rightward response trajectory (see Figure 1, panels A and B, dark blue arrows). Therefore, their results are consistent with the idea of a mental number line, a spatial representation of number on which small numbers are represented on the left and large numbers on the right (e.g. [4]). Indeed, if a correspondence is assumed between the position of a number on a mental number line and the position of a response in external space, then the results described by Song and Nakayama are to be expected. For example, the number 4 would elicit a more rightward response trajectory (Figure 1B, dark blue) than the number 1 (Figure 1A, dark blue), because 4 is located to the right of 1 on the number line. The assumption of a correspondence between the location of a number on the mental number line and the response location has been termed the *direct mapping account* [5].

An alternative interpretation is given by a computational model designed by Verguts, Fias and Stevens [6] to simulate the number comparison task. One of the goals of this model was to simulate the comparison distance effect, the observation that it is easier and faster to compare two numbers with a large distance (e.g., 1 and 9) than two numbers with a small distance (e.g., 5 and 6) [7]. In the Verguts et al. model [6], the numerical magnitude of the presented Arabic digits is represented by so-called number units. The available response alternatives (e.g. *left* and *right*) are instantiated by response units. Response time is determined by the amount of activity that the response units receive from the number units. When the distance between the two numbers is large, activation in the correct response unit rises quickly and there is little activation in the incorrect response unit. As a consequence, response times will be fast. In contrast, when the distance between the numbers is small, activation for the correct response unit rises more slowly and there is also some activation in the incorrect response unit. This results in a slower response time. Crucially, the decision is not transferred from “cognition” to “action”, but instead gradually takes shape at the response level. This is in line with the ecological perspective of Cisek and Kalaska [1]. Although the Verguts et al. model [6] was designed to simulate response time data and not response trajectories, it is natural to assume that the relative activation of the response units determines the reaching trajectory across time. If there is strong activation for one response and weak activation for the other, a straight response trajectory to the first response should occur. If there is a smaller difference between the activation of the two response units, we predict that the response trajectory towards the first response is more curved because of the influence of the second response unit. Intuitively, the second response unit “pulls the hand” toward its corresponding location. In this way, the findings by Song and Nakayama [3] can be accounted for. When there is a large distance between the numbers to be compared (e.g. when 1 has to be compared to the standard 5), there is a quick rise in activation in the correct response unit and only weak activation in the incorrect response unit. In this case, a straight trajectory to this response is to be expected. On the other hand, when the distance between numbers is small (e.g. when 4 has to be compared to the standard 5), activation in the correct response unit rises more slowly and there is also activation in the incorrect response unit. In this case, one predicts that the response trajectory is curved towards the other response. In summary, the Verguts et al. model [6] can account for the results of Song and Nakayama [3] because it predicts a distance effect in the movement trajectories, with more curved trajectories for smaller distances (see Figure 1, panels C and D, orange arrows).

Importantly for the present study, Song and Nakayama [3] only reported results for the response mapping in which the instructions were to reach for the left response location for a number smaller than 5 and to a right response location for a number larger than 5 (number line congruent mapping). With this response mapping, it is impossible to dissociate between the direct mapping account and the Verguts et al. account [6]. However, it is straightforward to deduce hypotheses from the direct mapping account for a reversed response mapping. With this number line incongruent response mapping, participants would have to reach to the left response location for numbers larger than 5 and to the right response locations for numbers smaller than 5. For the direct mapping account, smaller numbers would still elicit a more leftward response than larger numbers. For example, when responding to the number 1, the response trajectory would still be more leftwards than when responding to the number 4. Put differently, the deviation of the trajectory from the midline would be larger for 4 than for 1 (and also larger for 6 than for 9; see Figure 1, panels A and B, light blue arrows). The effect of numerical distance on the deviation from the midline observed with the number line congruent response mapping would thus be reversed when applying a number line incongruent response mapping. On the other hand, in the Verguts et al. model [6], the distance effect is independent of the response mapping. In other words, deviation of the response trajectory from the midline will always be smaller for numbers close to the standard than for numbers far from the standard (see Figure 1, panels C and D, orange arrows). In summary, both the Verguts et al. model and the direct mapping account predict the same pattern of response trajectories with a number line congruent response mapping (i.e. the mapping used in the experimental setup of Song & Nakayama [3]). In contrast, with a number line incongruent response mapping, the Verguts et al. model predicts more deviation from the midline with increasing distance between target and standard 5, while the direct mapping account predicts the opposite: less deviation from the midline with increasing distance. In the present study, these predictions are tested.

## Results

### Response Time and Accuracy

**Figure 2:**
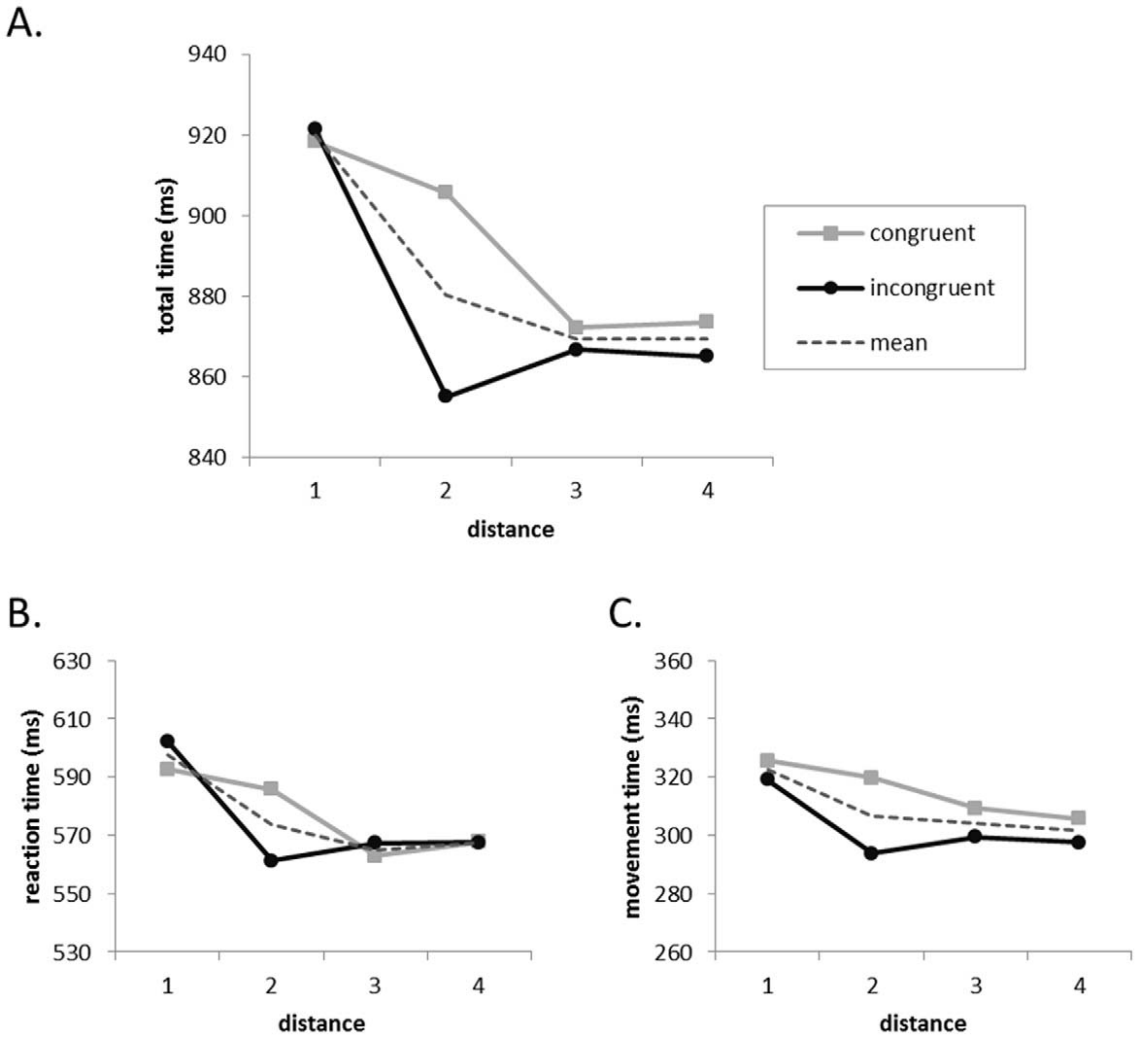
Response time analyses. The effect of numerical distance (1 to 4) and response mapping (number line congruent or incongruent and the mean of these two mappings) on total times (a), reaction times (b) and movement times (c). Note that the Y-axes have the same scale but a different range in the three panels.

*Total time* was defined as the interval between the onset of the stimulus presentation and the crossing with the index finger of one of the borders of the left or right square at the top of the screen. Like Song and Nakayama [3], we divided total time into two components: reaction time and movement time, although our operationalization of these measures differs slightly due to the technical specificities of this study. *Reaction time* was defined as the interval between stimulus (number) onset and the crossing of one of the borders of the start button. *Movement time* was defined as total time minus reaction time. Only correct trials entered the analysis, excluding trials on which the reaction time was less than 100 ms and the total time was more than 1500 ms (on average less than 1.5% of all correct trials). An ANOVA was run on the mean total times with a 4 (distance from the standard: 1 to 4) ×2 (response mapping: number line congruent or number line incongruent) design with both factors treated as within subjects variables. There was an effect of distance (F(3,42)  = 41.59; p<.001; 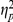
2 = .75) with decreasing total times for distances 1 to 4 (linear contrast: F(1,14) = 58.90; p<.001; see Figure 2A). The main effect of response mapping was not significant (F(1,14)<1; 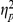 = .03). There was an interaction between distance and response mapping (F(3,42) = 11.72; p<.001; 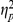 = .46). This effect seemed to be mainly driven by a faster reaction time for distance 2 with the number line incongruent mapping than with the number line congruent mapping (see Figure 2a). The second ANOVA had the same independent variables, but was computed on the mean reaction times. The results were very similar. There was a significant distance effect (F(3,42) = 26.79; p<.001; 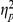 = .66), again showing a linear decrease with increasing distance (linear contrast: F(1,14) = 48.60; p<.001). There was no main effect of response mapping (F(1, 14)< 1; 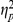
<.01). The interaction between distance and response mapping was significant (F(3,42) = 6.89; p<.001; 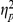 = .33) (see Figure 2b). A third, similar ANOVA was run on the mean movement times. They were again very similar, with a significant distance effect (F(3,42) = 12.84; p<.001; 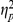 = .48) showing a linear decrease with increasing distance (linear contrast: F(1,14) = 19.51; p<.001). There was no significant effect of response mapping (F(1,14) <1; 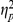 = .04). The interaction between distance and response mapping was significant (F(3,42) = 4.31; p<.05; 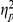 = .24) (see Figure 2c). Accuracy was above 99% for all participants and was therefore not analyzed.

### Movement Trajectories

**Figure 3:**
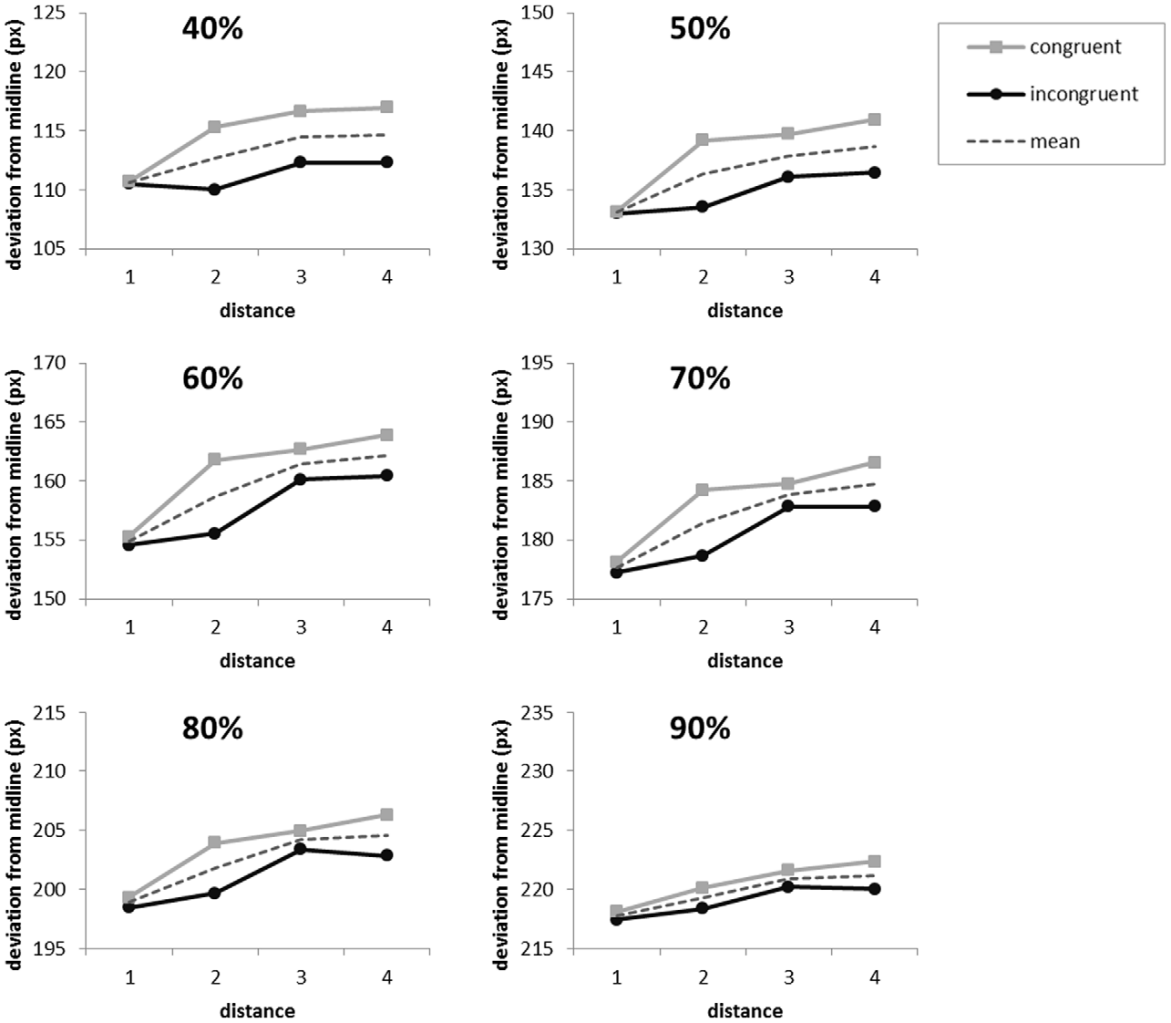
Response trajectory analyses. The effect of numerical distance (1 to 4) and response mapping (number line congruent or incongruent and the mean of these two mappings) on deviation from the midline in pixels. Only the slices for which a significant distance effect was found (40% to 90% of the trajectory) are plotted. Note that the Y-axes have the same scale but a different range for the different slices.

Similarly to Song and Nakayama [3], orthogonal distance of finger position to the midline (connecting start button and central top square) was computed separately for every trial at 10 equally spaced time points (10% to 100% of the movement time) by linear interpolation of the two nearest sampled data points. An ANOVA was run with a 4 (distance: 1 to 4) ×2 (response mapping: number line congruent or number line incongruent) design separately for every “slice” (10% to 100%) of the trajectory. Both factors were treated as within subjects variables. Trials that were excluded from the response time analyses were also excluded for the movement trajectory analyses. Significant distance effects (p<.05; 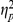 in the range between .194 and .517) were present from 40% to 90% of the trajectory: deviation from the midline increased with increasing distance. These distance effects all showed significant linear contrasts (p<.05). Importantly, there was no significant main effect of response mapping and no significant interaction between response mapping and distance in any of the slices of the trajectory (see Figure 3). As explained in the introduction, with the number line congruent mapping, both the direct mapping account and the Verguts et al. model [6] predict an increasing deviation from the midline with increasing distance. However, the number line incongruent mapping (move right when number is smaller than 5, left when number is larger than 5) allows distinguishing between the two theories. For this condition, the Verguts et al. model still predicts an increasing deviation from the midline with increasing distance, whereas the direct mapping account predicts the opposite effect: a decreasing deviation from the midline with increasing distance. To test this, we used planned comparisons to restrict the analyses specifically to the number line incongruent mapping. Like in the main analysis, the effect of distance was significant from 40% to 90% of the trajectory (p<.05; 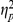 in the range between .267 and .415): deviation from the midline increased with increasing distance. These distance effects also showed significant linear contrasts (p<.05).

To exclude the possibility that participants were moving in a randomly chosen direction or along the midline during the first slices, we tested whether there was already a difference in moving towards the left versus moving towards the right response location at the very beginning of the movement trajectory (i.e. in the first slice). To this end, the left/right position of the finger relative to the midline was analyzed for the first slice. The t-test for the difference between the correct trials with a left target response and the correct trials with a right target response was significant (t(14)  = −14.89; p<.001). This indicates that participants were already moving towards the correct response at the very beginning of the movement trajectory.

## Discussion

We examined the trajectories of manual reaching for a response location during a number comparison task. We replicated the results of Song and Nakayama [3], showing that the smaller the distance from 5, the more response trajectories curve towards the incorrect (opposite) response. This finding could be interpreted either as a number line congruence (direct mapping account) or a distance effect in the trajectories (Verguts et al. model [6]). To disentangle these possibilities, we manipulated the response mapping within subjects. We showed that the distance effect in the response trajectory is independent of response mapping and that it remains significant in the number line incongruent mapping. This is incompatible with the assumption of a direct mapping between the position of a number on the mental number line and the spatial location of the response. In contrast, the distance effect in the movement trajectories can be explained by the Verguts et al. model [6]. In this model, activation in the correct response unit rises faster when the target is numerically more distant from the standard number. Therefore, a straighter response trajectory is expected with increasing distance from the standard number. If the distance from the standard number is small, there is less difference between the activation of the response units and therefore, a response trajectory that is more strongly curved towards the incorrect response can be expected.

Traditionally, the domain of numerical cognition has focused on how numbers are represented (perhaps on a mental number line, consistent with the direct mapping account outlined above). However, starting from computational models (e.g. [6], [8], [9]) an alternative view has emphasized how number representations interact with action. For example, manipulation of the response set required for a task [5], [10] or even how the responses are labeled [11] have generated effects that are hard or impossible to explain with a mental number line account. The present study adds important insights to the tight coupling of number representations (i.e. cognition) and response related processes (i.e. action): The fact that the distance effect is reflected in movement trajectories shows that we act before cognitive processing is finished. In one sense, this is obvious if we consider that in daily life, we constantly have to adapt our behavior to changing external and internal demands. However, the view that the time from stimulus presentation to response (i.e., the response time) can be decomposed into successive time intervals corresponding to discrete mental processing stages [12]–[14] has been highly influential in cognitive psychology. In the present study, we show that this view cannot hold: a typical signature of cognitive processing, the distance effect, was present in the response trajectories. As described in a review paper of Song and Nakayama [2], the same methodology has led to similar findings in many different domains. For example, response trajectories deflect towards the irrelevant stimulus location (left or right) in a Simon task, even when this stimulus location is on the opposite side of the correct response location [15]. In a semantic categorization task, response trajectories curve towards the response location corresponding to the competing category when an atypical exemplar of a category is presented [16]. Further, when subjects have to evaluate the truth (*yes* or *no*) of a proposition, response trajectories toward a *yes* response curve more towards a *no* response for decisions that are regarded as “less true” than others [17]. All these studies show that cognition and action evolve together in real time for the purpose of adaptive responding (here, complying with task instructions).

The idea of a tight coupling between perception, cognition and action is not new and can be traced back to the early days of experimental psychology when William James proposed his ideomotor theory [18]. However, even in recent years this idea has challenged established theories in diverse domains such as developmental psychology (e.g. [19]) or artificial intelligence (e.g. [20]). The current data suggest that even for highly abstract information such as number, purely cognitive accounts that focus on how this information is represented (e.g. on the mental number line) may have limited explanatory power. Instead, perception and action are equally important and tightly coupled with cognition. As a consequence, they should be incorporated when developing theoretical models of number processing.

## Materials and Methods

### Ethics Statement

The study was approved by the ethical committee of the Faculty of Psychology and Educational Sciences of Ghent University. All participants signed an informed consent prior to the experiment.

### Participants

Fifteen right-handed bachelor students from Ghent University received course credits to participate in the experiment. All participants had normal or corrected to normal vision.

### Stimuli and Apparatus

The experimental procedure was implemented using the Tscope library for the C programming language [21]. Response trajectories were tracked using a 15 inch touch screen, which also displayed the stimuli and the response locations. This allowed tracking the trajectories on the X and the Y axis. The start button was a square (length of sides was about 2.8 visual degrees) containing the word “START”, presented centrally at the bottom of the screen. At the top of the screen, three white squares (equal size as the start button) were displayed in a way similar to Song and Nakayama's setup. They were equally spaced horizontally at about 5.6 visual degrees from one another. The target stimulus, an Arabic digit, was always presented in the center square. The X and Y location on the touch screen were sampled at 100 Hz.

### Experimental Procedure

Participants performed a number comparison task on Arabic digits from 1 to 9, excluding 5. A trial started with the presentation of the start button at the bottom of the screen and three squares at the top of the screen. A fixation cross was displayed in the central top square as soon as the start button was touched. This cross was replaced by the target digit after an interval randomly drawn from a uniform distribution between 700 and 900 ms. Participants were asked to move as fast as possible to the left or the right square with their right index finger, while holding this finger on the screen. If they removed their index finger from the surface of the screen, a message on the screen after the trial instructed them to do so.

There were two experimental blocks of 200 trials each. In each block, every digit was thus presented 25 times. The order of the response mappings was counterbalanced between subjects: half of the participants started with moving from the start button to the left button if the digit was smaller than 5 and to the right button if the digit was larger than 5. The other half of the participants started by moving to the left button if the digit was larger than 5 and to the right button if it was smaller than 5. The response mapping was reversed after the first experimental block. To practice the response mapping, each experimental block was preceded by a practice block of 24 trials. Only in these practice blocks, negative feedback was given: after an incorrect response, the word “FOUT” (Dutch for “wrong”) was presented in red, centrally on screen for 800 ms. Before each practice block, the response mapping was displayed on the screen. Participants could take a short pause between blocks.
